# Usability and Acceptability of Two Smartphone Apps for Smoking Cessation Among Young Adults With Serious Mental Illness: Mixed Methods Study

**DOI:** 10.2196/26873

**Published:** 2021-07-07

**Authors:** Minda A Gowarty, Meghan R Longacre, Roger Vilardaga, Nathan J Kung, Ashley E Gaughan-Maher, Mary F Brunette

**Affiliations:** 1 Departments of Internal Medicine and Community and Family Medicine Dartmouth Hitchcock Medical Center Lebanon, NH United States; 2 Geisel School of Medicine at Dartmouth Hanover, NH United States; 3 Center for Technology and Behavioral Health Geisel School of Medicine at Dartmouth Lebanon, NH United States; 4 The Dartmouth Institute for Health Policy and Clinical Practice Geisel School of Medicine at Dartmouth Hanover, NH United States; 5 Department of Psychiatry and Behavioral Sciences Duke University School of Medicine Durham, NC United States; 6 Department of Psychiatry Dartmouth Hitchcock Medical Center Lebanon, NH United States

**Keywords:** smoking cessation, mHealth, serious mental illness, smartphone application, digital health, psychiatric illness, tobacco treatment, mobile phone

## Abstract

**Background:**

Young adults with serious mental illness (SMI) have higher smoking rates and lower cessation rates than young adults without SMI. Scalable interventions such as smartphone apps with evidence-based content (eg, the National Cancer Institute’s [NCI’s] QuitGuide and quitSTART) could increase access to potentially appealing and effective treatment for this group but have yet to be tested in this population.

**Objective:**

The goal of this user-centered design study is to determine the user experience (including usability and acceptability) of 2 widely available apps developed by the NCI—QuitGuide and quitSTART—among young adult tobacco users with SMI.

**Methods:**

We conducted usability and acceptability testing of QuitGuide and quitSTART among participants with SMI aged between 18 and 35 years who were stable in community mental health treatment between 2019 and 2020. Participants were randomly assigned to use QuitGuide or quitSTART on their smartphones. App usability was evaluated at baseline and following a 2-week field test of independent use via a video-recorded task completion protocol. Using a mixed method approach, we triangulated 4 data sources: nonparticipant observation, open-ended interviews, structured interviews (including the System Usability Scale [SUS]), and backend app use data obtained from the NCI. Quantitative data were analyzed using descriptive statistics, and qualitative data were analyzed using thematic analysis.

**Results:**

Participants were 17 smokers who were not interested in quitting, with a mean age of 29 (SD 4) years; 41% (n=7) presented with psychotic disorders. Participants smoked an average of 15 (SD 7) cigarettes per day. The mean SUS scores for QuitGuide were similar at visits one and two (mean 64, SD 18 and mean 66, SD 18, respectively). The mean SUS scores for quitSTART numerically increased from visit one (mean 55, SD 20) to visit two (mean 64, SD 16). Acceptability scores followed the same pattern. Observed task completion rates were at least 75% (7/9 for QuitGuide, 6/8 for quitSTART) for both apps at both visits for all but 2 tasks. During the 13-day trial period, QuitGuide and quitSTART users interacted with their assigned app on an average of 4.6 (SD 2.8) days versus 10.8 (SD 3.5) days, for a mean total of 5.6 (SD 3.8) interactions versus 41 (SD 26) interactions, and responded to a median of 1 notification (range 0-8) versus 18.5 notifications (range 0-37), respectively. Qualitative comments indicated moderate to high satisfaction overall but also included concerns about the accuracy of the apps’ feedback.

**Conclusions:**

Both QuitGuide and quitSTART had acceptable levels of usability and mixed levels of acceptability among young adults with SMI. The higher level of engagement with quitSTART suggests that quitSTART may be a favorable tool for young adult smokers with SMI. However, clinical support or coaching may be needed to overcome initial usability issues.

## Introduction

People with a serious mental illness (SMI), such as schizophrenia and severe mood or anxiety disorders, are more likely to smoke and less likely to quit than the general population [1-3]. Quitting before the age of 35 years may reverse the early mortality associated with smoking [4,5], providing an important rationale for engaging young smokers in cessation attempts. Although many studies have tested smoking cessation treatments in young adults in the general population [6,7], few studies have examined smoking cessation interventions in young adults with SMI [8,9].

Because of their widespread use and unique features, smartphone apps are a promising vehicle for smoking cessation interventions in people with SMI. Recent data demonstrate that nearly 80% of young adults with SMI use smartphones, and more than two-thirds are interested in using smartphones for health and wellness interventions [10]. Potential advantages of app interventions include the user’s ability to tailor their experience by entering personal data, access content on demand, be cued to practice a behavioral change skill, and receive personalized feedback on their progress [11]. Recent findings demonstrate that young adults with SMI value these and other app features, suggesting that apps may be well suited to deliver smoking cessation support to this population [12]. Although hundreds of smartphone apps are available for smoking cessation, they vary widely in their content and features [11,13-17]. To our knowledge, none have been evaluated for usability, appeal, or effectiveness in young adults with SMI.

The National Cancer Institute (NCI) provides 2 smoking cessation apps based on behavioral change theories and clinical practice guidelines [11,14-16,18,19]—one designed for adults (QuitGuide) and the other designed for teens (quitSTART). These apps vary considerably in their content, layout, and design. Research on previous versions of these apps (2013-2015) indicated superior content quality than most other available smoking cessation apps [14,16,19]. In addition to their content, the design and usability of digital tools affect their use with time and thus require considerable attention to ensure that an app will be accessed as intended by a user group. In particular, people with SMI have greater difficulty understanding abstract labels, navigating complex content configurations, and understanding content organization, which may deter the use of standard apps [14,20,21]. Testing of QuitGuide (and its precursor QuitPal) among middle-aged adults with SMI resulted in mixed usability reviews [14,22]. However, neither QuitGuide nor quitSTART has been tested in youth or young adults with SMI who grew up in an era of widespread mobile technology. As young adults generally report greater confidence and ease of use with technology than middle-aged adults [23], young adults with SMI may have a reasonable ability to use standard apps, despite cognitive limitations and other impediments.

Although there is increasing interest in using smartphone technology for behavioral smoking cessation interventions, early phase assessment of this technology offers crucial data in preparation for an efficacy trial [13]. Usability (“how well users can learn and use a product to achieve their goals”) and acceptability (which includes perceived value, usefulness, and desirability) are important components of user experience [24], and increased user engagement is associated with improved outcomes [25-27]. Given that the NCI’s apps are easily available and free, contain high-quality content, and provide numerous features of interest to young adults with SMI [12], we seek to evaluate the apps’ potential role for cessation interventions in this population by assessing their usability and appeal among young adults with SMI. We tested QuitGuide because we believe its simple design could be highly usable among young people with SMI, and we tested quitSTART because we believe its content and features could be more appealing than QuitGuide among young people with SMI.

## Methods

### Participants and Recruitment

Potentially eligible participants were recruited from a single large community mental health center in New England, United States, between May 2019 and February 2020 via flyers posted in waiting rooms and clinician invitations. Eligible participants were aged between 18 and 35 years, English speaking, stable in outpatient mental health treatment for SMI (ie, no hospitalization in the past 30 days per chart review), self-reported regular tobacco smokers (daily and nondaily) confirmed by breath carbon monoxide (CO)>7 parts per million (ppm) [28], and smartphone users (either Apple or Android). The desire to quit smoking was not required. We excluded patients who were pregnant or had a current, unstable substance use disorder per chart review or the patient’s mental health center clinician. We aimed to recruit 5 participants with psychotic disorders and 5 with other SMI diagnoses per app, as prior usability research has demonstrated that more than 80% of usability issues can be identified after the first 5 participants [29].

### Interventions

The QuitGuide and quitSTART apps are available free of charge on Smokefree.gov via the Apple Store or Google Play. Both apps encourage the user to set a quit date within 14 days, provide information about quitting, and allow users to enter personal data, such as how many cigarettes they smoked per day, what times of day they tend to smoke, and how much they spend per pack of cigarettes. They allow users to track cigarette cravings, *slips* (defined as cigarettes smoked for this study), and moods and provide information regarding coping with these experiences. They also provide information on users’ progress, such as cigarettes avoided and dollars saved by not smoking. In addition, users can connect to social media through the apps.

**Figure 1:**
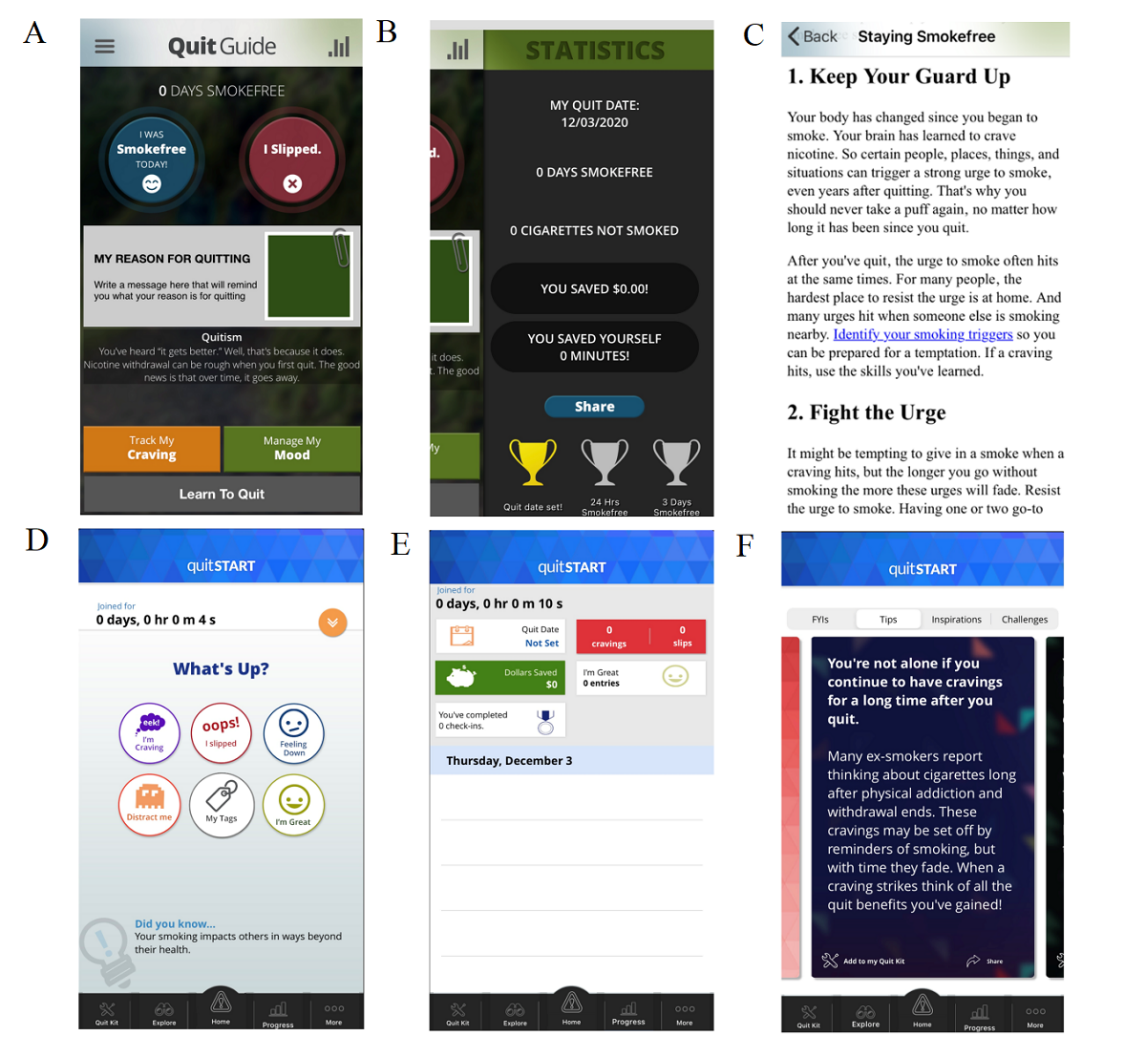
Selected screenshots from QuitGuide (A-C) and quitSTART (D-F).

Although QuitGuide and quitSTART offer many similar features, their design and content differ in a number of ways (Figure 1). QuitGuide offers a relatively linear layout, utilizes darker colors that convey a serious tone, and provides information through text with minimal graphics. Users can type journal entries and can read a text-only guide on how to quit smoking. In contrast, quitSTART utilizes a more complex layout with bright colors and informal language that results in a cheery tone and prominently displays relatively large symbols and few words within the icons. Information in quitSTART is displayed on swipeable *cards*, each with a different color background and 5 or fewer sentences. quitSTART provides a selection of 7 games for distraction that can be played within the app. Both apps allow users to set notifications based on time or location, and quitSTART also automatically sends check-in notifications that ask users how many cigarettes they have smoked since the last check-in (QuitGuide does not have an analogous check-in feature).

### Procedures

Potentially eligible participants completed an informed consent process and proceeded with the study procedures once eligibility was confirmed. The first 12 participants received a US $30 gift card to a retail store after completing each of the 2 study visits (for a total of US $60). To improve recruitment, we increased compensation to US $60 per visit (for a total of US $120) for the last 5 participants. The New Hampshire State Institutional Review Board approved and monitored all study activities.

Participation in the study lasted for 2 weeks. A trained researcher obtained demographics and diagnoses from record reviews and conducted structured interview assessments using a standardized procedure in which measures were administered aloud and answers were recorded on paper forms and then entered immediately into a computerized database. To assist with answering multiple-choice questions, answer choices were provided as visual cues on paper. A component of the assessment included a semistructured, open-ended interview, which was audio-recorded. Usability tasks were video-recorded.

At the first study visit (visit 1), participants completed a structured interview assessing demographics, tobacco use, and technology use. Participants were then randomly assigned to 1 of the 2 apps (QuitGuide or quitSTART) in groups of 4 blocked by age group [30]. They were asked to download the app on their smartphones. Researchers oriented the participants to the *think-aloud* method [31] and video-recorded participants as they completed a set of 9 predefined tasks without researcher guidance. Researchers then assessed participants’ perceived acceptability and ease of use of their assigned app using the measures described below.

At the conclusion of the first study visit, we provided participants with a brief in-person tutorial on using their assigned app. The tutorial focused on helping users obtain skills for 3 tasks within the app: setting a quit date, logging and viewing logged moods, and logging and viewing logged *slips*; these tasks were chosen based on guideline-recommended interventions [32], the importance of mental health symptoms to smoking in this population [33], and our prior findings that young adults with SMI want to track cigarettes [12], respectively. We instructed participants to use the app independently over the following two-week period. We recommended that they try to skip cigarettes using their assigned app, but we did not advise participants to quit smoking for this study.

Following this period of independent use, participants returned for a second study visit (visit 2) and were assessed for smoking, perceptions of acceptability and usability, and task performance within the usability task protocol.

### Measures

#### Demographics, Technology Use, and Diagnosis

Using a structured interview, researchers obtained participants’ demographics at visit 1 and history of technology use (eg, frequency of internet use and app use) at visits 1 and 2. Psychiatric diagnosis and stability—as determined by mental health center clinicians—as well as insurance information were obtained via medical chart review at visit 1.

#### Tobacco History and Smoking

Researchers obtained participants’ history of tobacco use (eg, duration and frequency of smoking, product use, and prior quit attempts) with a structured interview at visit 1. At visits 1 and 2, we assessed tobacco dependence using the Fagerström test for nicotine dependence [34], a 6-item scale shown to be reliable and valid among smokers with SMI [35]. We obtained smoking status (yes or no) and confirmed this via exhaled breath CO>7 ppm (measured with a Covita Smokerlyzer) at both visits [28].

#### App Feature Preferences

Before performing the usability protocol, participants were asked to rate 15 app features on a 5-point Likert-type scale according to how important they believed the features were to help someone quit or reduce their smoking (Multimedia Appendix 1). The researchers chose the features included in this task based on clinical practice guidelines as well as prior studies reporting users’ preferences within smoking cessation apps [32,36,37].

#### Observed App Usability

The following usability protocol was developed and administered following a user-centered design methodology [24,38]. Participants were oriented to the *think-aloud* procedure [31], after which they were given up to 5 minutes to freely explore the app while practicing thinking aloud. Participants were then asked to complete 9 specified tasks within the app (Multimedia Appendix 2) while thinking aloud and were provided as much time as they felt necessary to complete each task before moving on to the next task. Tasks included setting a quit date (Quitdate), reporting how many cigarettes they smoke per day (CigsperDay), logging a good mood (FeelingGood), logging a craving (Craving), finding information on how to quit smoking (HowtoQuit), logging a *slip* (smoked cigarette; Slipped), finding the progress page (Progress), connecting to social media (SocialMedia), and uploading a photo (Photo). Tasks were chosen based on US Clinical Practice Guidelines [32] as well as prior studies that evaluated frequently used features, desired app features, and features that have been correlated with point prevalence abstinence [12,36,37]. The participants’ phone screens and hand motions were video-recorded as they completed the tasks.

The video recordings were scored as follows: a task was designated *completed* if the participant was able to reach the requested end point, regardless of whether they encountered difficulties along the way. A task was designated as *not completed* if the participant requested to skip the task or indicated that they had completed the task but did not reach the requested end point. *Usability challenges* were defined either as actions performed in the app that could not be used to reach the requested end point or difficulty reaching the requested end point identified either by researcher review or by participant verbalization during the task.

#### App Perceptions Qualitative Interview

At each visit, we conducted and audio recorded a brief, semistructured, open-ended qualitative interview to assess perceived ease of use and acceptability of the apps. During the first visit, interview questions assessed participants’ general feedback about their assigned app, including likes and dislikes, and recommendations for changes to the apps’ features, graphics, or layout. During the second visit, these questions were repeated with additional questions regarding app features, such as cigarette tracking and notifications.

#### System Usability Scale

The System Usability Scale (SUS) [39] is a validated questionnaire widely used to assess the usability of various technologies [22,40,41]. Scores range from 0 to 100, with values between 68 and 70 representing the average usability [42,43].

#### Perceived Ease of Use and Acceptability Questionnaire

A 14-item questionnaire assessed perceived ease of use and general acceptability of the apps, comprising a subset of questions derived from the Post-Study System Usability Questionnaire [44] and the Usefulness, Satisfaction, and Ease of Use Questionnaire [45]. We chose a subset of questions from these scales that have been previously used in people with SMI [46] and used a 5-point Likert-type scale for consistency among our study questions.

#### App Utilization

The NCI provided backend app usage data, including date and time of app use, features activated in the apps, and responses to notifications [47]. An app interaction was defined as the user opening the app and activating at least one feature (whether or not a notification from the app prompted this), with interaction instances separated by at least 25 minutes. A cutoff of 25 minutes was chosen to avoid interpreting prolonged interaction with one feature (such as reading the *How to Quit* section in QuitGuide or playing a game in quitSTART) before engaging with another feature as more than one episode of engagement with the app.

#### Participant Flow

We identified 98 potential candidates for inclusion in this study. Overall, 35% (34/98) potential candidates were ineligible based on prescreening criteria, 7% (7/98) were unable to participate because of time constraints related to work or childcare responsibilities, 1% (1/98) was in the process of moving to another location, 10% (10/98) did not have working smartphones, and 28% (27/98) declined to participate. The remaining 19% (19/98) individuals provided informed consent. Of these, 2% (2/98) were ultimately deemed ineligible because of breath CO below the cutoff for inclusion. Thus, 17% (17/98) participants were included in the study. All 17 participants completed visits 1 and 2 (100% retention). Backend app usage data from the 2-week trial period were available for 15 out of 17 participants (88%; home app use data were not available for 2 quitSTART participants because of issues with the participants’ phones, and these participants were excluded from the app utilization analyses).

### Data Analysis

#### Quantitative Analyses

Descriptive statistics were used for all quantitative analyses; 1 usability task was missing for 1 participant at visit 1. These data were omitted from the analysis. For the 15 participants with available backend app usage data, we analyzed home app use on days 2 to 14 (full days with the opportunity for app use during the entire day). The first and last days of app use were excluded because participants completed the usability tasks on those days. Complete data were available for all 15 participants on all days except day 14 as a participant was assessed a day earlier.

Video recordings from the usability task completion protocol were reviewed to assess the participants’ ability to reach the prespecified end point for each task and identify difficulties encountered during task completion. Participant navigation through the apps was compared with maps of each app created by the research team to determine task completion rates and usability challenges. Researchers also included participants’ comments during the session regarding their intended navigation through the apps to further assess usability challenges. During the initial coding of the videos, definitions regarding usability challenges were refined until a final set of definitions was reached. The final coding of the video recordings was performed using this set of definitions.

#### Qualitative Analyses

Audio recordings of the qualitative responses to the semistructured interview questions were transcribed and compared with the original audio files to ensure accuracy. The transcripts were analyzed using thematic analytical techniques [48]. After conducting an immersive review of the data set, 3 researchers (MAG, NJK, and AEM) independently applied structural and inductive coding methodologies [49] to each of the interview transcripts using either Microsoft Word (Microsoft Corporation) or the qualitative data analysis program, Atlas.ti (Version 8, ATLAS.ti Scientific Software Development GmbH). Because of the descriptive nature of the data, themes naturally emerged during the initial coding process, and researchers reached a consensus regarding these themes after a single discussion. Negative case analysis was used to ensure that the entire data set was represented in the emerging themes.

## Results

### Participant Characteristics

As shown in Table 1, participants were 17 daily smokers with a mean age of 29 (SD 4) years, 41% (7/17) were diagnosed with psychotic disorders, and 94% (16/17) were Medicare or Medicaid beneficiaries (Table 1). Participants smoked an average of 15 (SD 7) cigarettes per day and were moderately dependent on tobacco (mean Fagerström score 4.4, SD 1.8). More than 90% (16/17) of participants reported using smartphone apps on a daily basis, and more than 75% (13/17) had previously downloaded an app related to health and wellness. Of the 17 participants, only 4 (24%) endorsed previously trying a smartphone app to aid in a quit smoking attempt; of the remaining 13 participants, 11 (85%) were unaware that smartphone apps were available to help people quit smoking.

**Table 1:** Participant characteristics (N=17).

Characteristic		Values
**Demographic and clinical characteristics**		
	Age (years), mean (SD)	29 (4)
	Female, n (%)	7 (41)
	White, n (%)	16 (94)
	High school diploma, n (%)	14 (82)
	Psychotic disorder, n (%)	7 (41)
	Currently employed (part-time or full-time), n (%)	8 (47)
	Medicaid or Medicare beneficiary, n (%)	16 (94)
**Tobacco use characteristics**		
	Cigarettes per day, mean (SD)	15 (7)
	Baseline breath carbon monoxide, mean (SD)	26 (11)
	Fagerström score, mean (SD)	4.4 (1.8)
	Age started smoking, mean (SD)	13 (3.5)
	Previous quit attempt, n (%)	15 (88)
**Smartphone use characteristics, n (%)**		
	Use smartphone ≥twice daily	16 (94)
	Use apps at least once per day	16 (94)
	Ever downloaded a health app	13 (77)
	Would try app if recommended by a doctor	14 (82)

### Appeal of App Features

A majority of participants agreed or strongly agreed at both visits that most of the proposed app features were important to help someone quit smoking (Multimedia Appendix 3). The importance of location tracking increased from 35% (6/17) of participants at visit 1 to 59% (10/17) at visit 2, whereas the importance of tracking smoking triggers decreased from 71% (12/17) to 47% (8/17). Less than half of the participants indicated that sharing progress on social media was important: 41% (7/17) at both visits.

### Usability

The mean SUS scores for QuitGuide were similar at visits 1 and 2 (64; range 30-77.5, SD 18, and 66; range 25-85, SD 18, respectively). In contrast, mean SUS scores for quitSTART numerically increased from visit 1 (55; range 25-82.5, SD 20) to visit 2 (64; range 35-85, SD 16). Responses to the ease of use questions followed a similar pattern (Table 2). By the second visit, at least three-quarters of both QuitGuide and quitSTART users reported feeling satisfied with their app’s ease of use. In general, QuitGuide’s ease of use question responses were similar at visits 1 and 2, whereas affirmative responses to most questions regarding quitSTART’s ease of use increased between visits 1 and 2.

**Figure 2:**
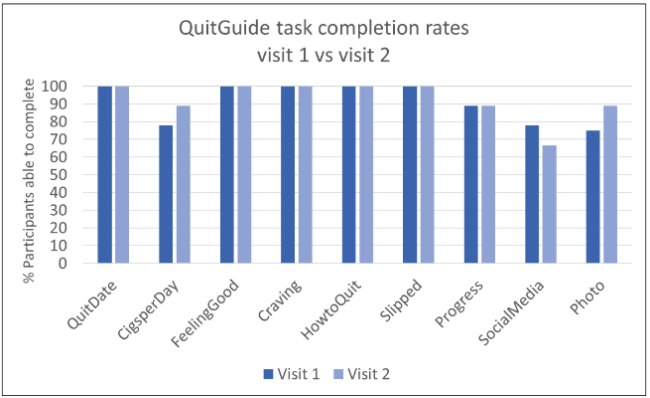
QuitGuide task completion rates.

**Figure 3:**
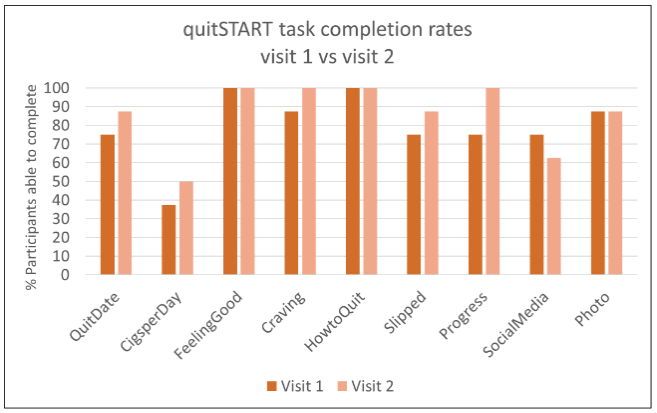
quitSTART task completion rates.

The observed usability task completion rates for both apps were high at both visits (Figures 2 and 3). At the first study visit, all 9 tasks were successfully completed by at least 75% of participants assigned to QuitGuide (7/9 for the first 8 tasks and 6/8 for the final task); similarly, 8 of the 9 tasks were successfully completed at the first study visit by at least 75% (6/8) of participants assigned to quitSTART.

**Table 2:** Ease of use and acceptability questionnaire results.

Statement^a^			QuitGuide (n=9)					quitSTART (n=8)		
			Visit 1, n (%)		Visit 2, n (%)		Visit 1, n (%)			Visit 2, n (%)
**Ease of use**										
	Overall, I am satisfied with how easy it is to use the app	7 (78)		7 (78)		4 (50)			6 (75)	
	I felt comfortable using the app	5 (56)		6 (67)		4 (50)			6 (75)	
	It was easy to learn to use the app	7 (78)		8 (89)		2 (25)			5 (63)	
	Whenever I made a mistake using the app, I could recover quickly and easily	8 (89)		6 (67)		5 (63)			4 (50)	
	It was easy to find the information I needed	6 (67)		7 (78)		1 (13)			5 (63)	
	How things appeared on the screen was clear	7 (78)		7 (78)		4 (50)			5 (63)	
**Acceptability**										
	Overall, I am satisfied with the app	6 (67)		5 (56)		4 (50)			5 (63)	
	I liked using the app	5 (56)		5 (56)		2 (25)			5 (63)	
	The app has all the functions and capabilities I expect it to have	6 (67)		4 (44)		3 (38)			2 (25)	
	I would recommend the app to a friend	5 (56)		7 (78)		3 (38)			5 (63)	
	The app is fun to use	4 (44)		4 (44)		3 (38)			2 (25)	
	The app works the way I want it to	4 (44)		4 (44)		2 (25)			1 (13)	
	The app can help me quit smoking	5 (56)		6 (67)		2 (25)			5 (63)	
	The app was interactive enough	6 (67)		5 (56)		4 (50)			5 (63)	

^a^Percentage of participants who agree or strongly agree with the corresponding statements.

The most common usability challenges occurred during attempts at 4 tasks: entering the number of cigarettes smoked per day, setting a quit date, connecting to social media, and uploading a photo. The first 3 of these tasks required navigation through menus, whereas the remaining tasks we assessed could be reached with a single click from the home screen, suggesting that menu navigation was associated with lower usability. The photo feature in QuitGuide (but not quitSTART) could also be accessed from the home screen but was uniquely challenging in that it only intermittently opened when users clicked on it because of a bug in the app (which has since been fixed).

Some feature locations were not intuitive to the participants. Users could enter their quit date during the initial app setup and later in their Quit Plan (QuitGuide) or Profile (quitSTART). Additionally, the apps were not designed to track cigarettes smoked on a daily basis but asked users to enter the average cigarettes smoked per day in the same locations (ie, Quit Plan or Profile). Many participants tried to enter cigarettes smoked or change their quit dates on the progress pages, but these pages were designed only for viewing information and not entering information. This sometimes led to participant frustration after repeated attempts to click on the pages’ inactive icons. Although some participants struggled with this issue, others were able to complete these tasks by accessing their Quit Plan in QuitGuide or their Profile in quitSTART.

Notably, setting a quit date required the user to set a date within the next 14 days. Although most users were ultimately able to find where to set a quit date, they were not planning to quit during this time frame, and the apps did not allow them to enter a later date (although quitSTART did include a *not ready* option). Both apps recommended users to choose a quit date within the next 14 days; however, most participants did not see this explanation located above the date selection field, and many users became confused when the apps defaulted to 14 days from the current date. They allowed the app to choose a quit date for them, even though they verbalized that the selected date was an unrealistic goal that they could not achieve.

Common menu navigation challenges involved misunderstanding menu labels and expecting to find features in certain locations in the app based on prior experience with other apps. A commonly misunderstood menu label was *My tags* for both apps. Although the intended meaning was to identify (ie, *tag*) the times and locations when participants were at a higher risk of smoking, many participants interpreted this label to indicate a connection to social media. Other tasks, such as uploading a photo in QuitGuide, proved problematic when participants tried to use their experience with other apps to guide them. For example, many participants expected to find this option in the Settings feature of the app, though the Settings feature does not contain this option. Instead, the photo feature could be accessed within the Quit Plan under *Reasons to Be Smokefree*. In contrast, quitSTART users were able to upload a photo in the Profile section of the app (QuitGuide does not have a Profile feature).

Finally, participants often used personal information (data) entry pathways to complete tasks unrelated to logging or tracking personal information in the app, potentially leading to inaccurate feedback to the user if the app tailors feedback based on these features. For example, users can obtain information about quitting in both apps by touching the *slip* button on the home page, and both apps provide a tally of the user’s entered slips on the Progress page. Participants in both groups frequently used the slip feature to obtain information about quitting, often favoring it over alternate pathways that involved more complicated menu navigation (which did not involve data entry). Although they were ultimately able to reach the desired end point, using these pathways when not planning to log information influences feedback on the Progress page, which users perceive as being inaccurate as discussed below.

### Acceptability

Responses to the acceptability questions are presented in Table 2. Notably, more than half (5/9, 56%) of QuitGuide users indicated that they liked using the app at both visits, whereas the proportion of users who liked using quitSTART increased from 25% (2/8) at visit 1 to 63% (5/8) at visit 2. About two-thirds of users thought each app would help them quit smoking at the second study visit, although fewer participants in each group felt that their assigned app had all the functions they expected it to have at visit 2 compared with visit 1.

### App Utilization

**Figure 4:**
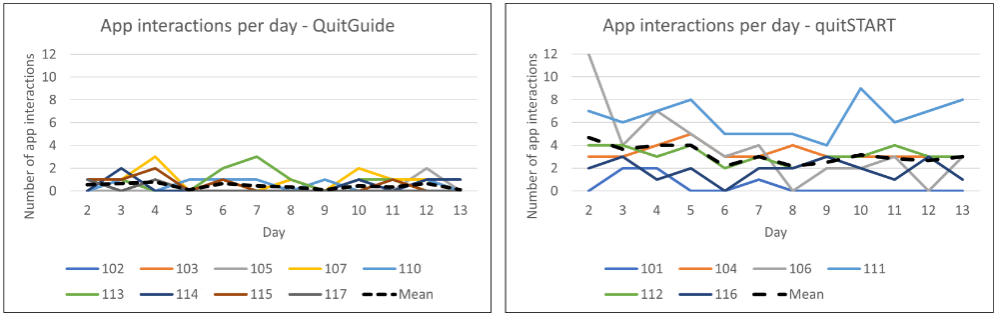
App interactions per day by participant for QuitGuide and quitSTART.

In contrast to the SUS scores, app use patterns demonstrated dramatically greater engagement with quitSTART than QuitGuide (Figure 4). Compared with QuitGuide users, quitSTART users demonstrated greater mean days of use (10.8, SD 3.5, vs 4.6, SD 2.8), greater mean total app interactions (41, SD 26, vs 5.6, SD 3.8), and greater median responses to notifications (18.5, range 0-37, vs 1, range 0-8) during the 13-day period.

### Tobacco Use at Follow-Up

At visit 2, 78% (7/9) QuitGuide users reported that they had tried to quit or cut back during the 2-week trial period (mean CO for all QuitGuide users 25, SD 12); no participants quit. Similarly, 75% (6/8) of quitSTART users reported trying to quit or cut back during the study (mean CO for all quitSTART users 25, SD 16). In fact, 25% (2/8) of quitSTART users reported that they no longer smoked at visit 2 (both confirmed with breath CO <7 ppm), having instead switched completely to e-cigarettes.

### Qualitative Feedback and User Experience

Themes resulting from qualitative data analysis of QuitGuide interview data conveyed a mildly positive view of the app. In contrast, the themes among quitSTART users included stronger reactions that were both positive and negative. A major theme among QuitGuide users at both visits was that the app was easy to use; although a minor theme, participants found some aspects of navigation confusing. Among quitSTART users, difficult navigation was a theme during the first visit, but ease of use was a stronger theme during the second visit.

The most prominent theme at visit 1 was the same for both apps: participants liked that the apps used a positive and supportive tone and provided motivational quotes and feedback on money saved. They thought that the apps could track cigarettes smoked on a daily basis and liked the idea of that feature. Most quitSTART users also expressed interest in the games included within the quitSTART app at visit 1 (QuitGuide does not offer games).

At visit 2, many participants continued to perceive both apps as positive and supportive and noted that this was a strength of the apps. Many participants voiced a general concern that a negative tone or repeated reminders of a lack of progress would evoke feelings of guilt and failure, which could undermine their quit attempts. Although some participants worried that calling a smoked cigarette a *slip* in these apps could evoke negative emotions, most participants in both groups commented on the overall positive tone of the apps and how this was a necessary attribute to maintain their engagement over time. In addition, a strong theme was feeling cared for by the apps. A quitSTART user, who was initially very skeptical about using a smartphone app to quit smoking, commented on quitSTART’s check-ins at visit 2:

You know, it’s nice. Like ‘Oh, okay, maybe someone cares out there’.

Participant 108

Similarly, a QuitGuide user noted:

And it’s good to have something looking out for you and asking you how you feel. It makes you feel, like, a little better.

Participant 105

Another strong theme for users of both apps was the importance of notifications at the second study visit. QuitGuide users wished that they had received more notifications, as they often forgot to open the app. quitSTART users often mentioned that the *check-in* notification feature was one of the most valued app features because it reminded them to use the app and increased their awareness of their smoking and because they appreciated the caring tone of these notifications. In fact, many quitSTART users (who received multiple notifications per day) indicated that they wished that they had received more.

In addition to noting the strengths of the apps at visit 2, a strong theme among participants in both groups was frustration at how the feedback features ultimately functioned for them. A quitSTART participant noted:

It kept giving me badges that I didn’t do...It gave me one at seven days smoke free, which I wasn’t, even though I was trying not to smoke.

Participant 106

Similar sentiments were expressed by QuitGuide users:

...I believe it says there’s, like, 14 days without a cigarette, but I was writing that I slipped up like it said, and it wasn’t correcting it.

Participant 103

Some of this app feedback relied on the users’ entered quit dates and could be updated by choosing a new quit date. However, only some user data reset when a new quit date is chosen, which further confuses some participants. In addition, some of the calculations were mathematically incorrect, possibly because of a bug in the app.

Many participants in both groups also expressed a desire for a cigarette tracking feature that enabled them to track cutting down, which they felt was important to frame their progress positively. Instead of tracking *slips* or smoke-free days, they expressed a desire for a daily cigarette tally, so they could track incremental progress toward quitting. A QuitGuide user stated:

You know, this day I’m only gonna smoke this many cigarettes and track each cigarette I smoked. Because I felt like that would be less of me failing, and more like the app helping me be able to see, like, ‘Hey today you smoked twenty. Yesterday you smoked twenty-five. Good job, you cut a couple out.’

Participant 102

Participants in both groups also wished that there were more sections in the apps where they could enter free-text responses to prompts (such as their moods or their triggers for smoking) instead of choosing from a prepopulated menu. As a participant noted:

There’s gotta be, like, a write your own response of why you slipped if you slipped. You know, if you wanna try to track what’s causing you to smoke, you can’t have just ten preset answers. There’s so much more to life than that.

Participant 102

Notably, most participants had little desire to connect with others on social media about their quit attempts. They worried that sharing information about quitting on social media could be detrimental if they were not successful in their quit attempts. In contrast, many suggested that the apps include a chat feature to connect with other app users. They felt that social support from others who were working on quitting, and therefore understood the challenge of quitting, could be helpful.

Participants provided opposing opinions regarding many of the remaining app features, highlighting the importance of a personalized experience for each user. Some participants planned to use only 1 or 2 app features, whereas others indicated their intention to explore all of the different features within their assigned app. Although many participants stated that they would not use certain app features, they also commonly recommended against removing these features from the apps because they thought other users might find them helpful. For example, in describing the games in quitSTART, a user stated:

...like, I would hate for them not to be there, but I just didn’t play them.

Participant 106

These young adult users of quitSTART made no comments indicating that they thought it was designed for teens and not for them.

## Discussion

### Principal Findings

In this user-centered design study, we used a mixed methods approach and triangulated multiple measures to assess usability and appeal. We found that QuitGuide demonstrated greater initial ease of use and acceptability, which remained stable over time. In contrast, quitSTART demonstrated lower initial usability and acceptability, which improved over time to a level similar to that of QuitGuide. Although SUS scores indicated below average usability for both apps (*average* score between 68 and 70 [42,43]), the objective quantitative and qualitative usability measures provided positive indications of usability. First, objective task completion demonstrated that at least three-quarters of participants were able to complete all but one task upon first downloading the apps. Second, during the open-ended qualitative interviews, participants stated that the apps were easy to use (at both visits for QuitGuide, and primarily at the follow-up visit for quitSTART). Several other measures also suggested that quitSTART performed well and provided value to users. First, the backend administrative data analyses showed that although user engagement with QuitGuide remained low during the trial period, quitSTART users sustained a substantially higher level of engagement (discussed in detail below). Second, although none of the users were required to engage in a quit attempt during this study, 2 of the 8 quitSTART users had biologically confirmed abstinence from smoking at follow-up compared with none of the QuitGuide users. Notably, although quitSTART was designed for teens, by Visit 2 it was perceived positively based on the qualitative feedback and a high level of engagement among these young adults with SMI. Given the acceptable perceived usability at the second visit, the much higher level of engagement with quitSTART, and previous work demonstrating the importance of engagement to cessation outcomes [25-27], our findings suggest that quitSTART may be a reasonable choice for use among young adult smokers with SMI, particularly if support and coaching facilitate initial use of the app.

A strong behavioral indicator of usability and acceptability is engagement over time. One of the most striking differences we found between the apps was the participants’ engagement, or frequency of use, during the 2-week trial period. Engagement with quitSTART, 2 to 4 interactions per day that persisted steadily over the 2 weeks, was much more favorable than engagement with QuitGuide (less than 1 per day). On the basis of participant feedback, app notifications played an important role in the different use patterns of the apps. Although some participants initially voiced concern about receiving too many notifications, many ultimately felt that notifications were positive and important to their engagement with the apps. Because our participants were not required to wish to quit smoking and were not engaged in a cessation program, the level of engagement here is likely lower than what would be seen among smokers trying to quit.

Notably, although both apps contain content and features that participants deemed important and desirable during a quit attempt, the overall appeal of the apps was influenced by perceptions of the app’s tone and data tracking. Previous work [50-53] has documented the importance of positive message framing to engage tobacco users in considering a quit or reduction attempt, and our findings further support this. In addition, our participants noted the importance of a positive tone within the apps to support ongoing motivation and indicated that a negative tone could undermine their quit or reduction attempts. Users in both groups felt that the apps were overall positive and motivating and indicated that this was a key factor in their interest in using the apps.

Not surprisingly, the perceived inaccuracy of the feedback (such as the number of slips or money saved) had a significant impact on users’ overall perceptions of the apps. Some of the perceived inaccuracy was because of the apps’ reliance on the entered quit date to calculate money saved and cigarettes avoided. Our participants were not required to engage in a quit attempt for this study, and some of the perceived data inaccuracy was likely because of choosing a default date without intending to quit on that day. Although the apps may be intended for users who plan to quit abruptly, our participants expressed interest in using the apps within a reduction-to-quit framework [54]. Incorporation of features within the app to support initial smoking reduction followed by cessation may be beneficial for this population. In addition, our findings indicate that entering personal information to track progress should be unlinked from other app features such as viewing inspirational quotes; otherwise, accessing these other features could also affect the accuracy of the users’ feedback.

### Comparison With Previous Work

Compared with recently reported data among middle-aged adults with SMI who were trying to quit smoking, QuitGuide usability scores were lower among these young adult participants [41]. Among middle-aged adults with SMI, the mean SUS score for young adults was 78.4 (SD 16.5), compared with 64 (SD 18) in this study. We are not aware of other published usability studies of the QuitGuide app among young adults with SMI.

Comparison of user engagement data among studies can be challenging because of different durations of follow-up, varied measurements of app engagement (eg, app openings, days of use, and specific actions within the app), and previously demonstrated decay in the use of eHealth interventions over time [25]. Nevertheless, our QuitGuide findings appear similar to those from other studies. In the study of middle-aged adults with SMI by Vilardaga et al [41], participants used the app on 32 (SD 24.5) days during the 120-day trial period, whereas the young adults in this study used the app on 4.6 days during our 13-day study period. Bricker et al [55,56] have previously assessed QuitGuide as a comparator app for evaluating novel cessation apps among middle-aged general population smokers trying to quit. In one trial [55], QuitGuide users self-reported opening the app an average of 15 times during an 8-week study period (days of use not reported). In another study [56], backend app usage data demonstrated that QuitGuide users opened the app 9.9 times on 7.1 days during a 12-month trial period. Satisfaction with QuitGuide ranged from 45%-70% in these trials. Hebert et al [57] have also assessed QuitGuide as a comparator for a just-in-time adaptive intervention. In this pilot study, QuitGuide users opened the app an average of 9.9 times on an average of 10.6 days during a 5-week trial period. Satisfaction scores for QuitGuide were lower than for the just-in-time intervention or usual care (in-person and/or telephone counseling), with QuitGuide averaging 3.64/5 for the survey item “I believe that my treatment will help me quit smoking and stay quit.”

Although we were unable to identify other studies that assessed the acceptability, usability, or user engagement of quitSTART, our findings regarding user engagement and satisfaction with quitSTART are promising compared with those of other apps in the general population of adult tobacco users. This includes studies of SmartQuit [55], in which users opened the app an average of 37 times during the 8-week trial period (days of use not reported), and of whom 59% were satisfied with SmartQuit overall; iCanQuit [56], in which users opened the app an average of 37.5 times on an average of 24.3 days during the 12 month trial period, and more than 80% of iCanQuit users found their app useful for quitting; and Clickotine [58], in which users opened the app an average of 100.6 times during the 8-week study period (days of use not reported).

Our qualitative usability findings are similar to those of Ferron et al [14], which found that middle-aged adults with SMI noted text-heavy apps to be unappealing, had difficulty navigating more engaging apps because of abstract symbols and one-word menu labels, and had difficulty following subtle directions to use various app features. Notably, our young adult participants rapidly overcame most challenges with these design features in quitSTART after a brief coaching session and 2 weeks of independent use.

Our acceptability findings are similar to those of other evaluations of middle-aged adults with SMI [40,59]. The evaluation of an earlier version of QuitGuide (QuitPal) by Vilardaga et al [40] highlighted participants’ desire for finer-grained cigarette tracking and interactive and motivating features, as well as the importance of seeing incremental progress. Klein et al [59] found that middle-aged participants with SMI expressed the importance of social support within the app, the role of caring and positivity from the app, and concern for negative emotions related to relapse. Our findings also significantly overlap with the assessment of Struik et al [60] among general population young adult tobacco users who assessed the Crush the Crave app, including the importance of positive message framing, preference for lighter colors, and frustration that progress feedback based on the user’s quit date was not accurate.

### Limitations

Our study has several limitations. First, we included a small number of participants because prior research has demonstrated the adequacy of this number for identifying most usability issues [29], but our acceptability findings should be interpreted with caution. Second, participants were not required to be interested in quitting or engaging in a quit attempt for this study. App preferences may differ during planned quit attempts when engagement is likely to be higher. Although more than three-quarters of our participants reported attempting to quit or reduce their smoking during the trial period, they had not committed to cessation treatment. This may have contributed to the frustration with inaccurate feedback, which was based on the entered quit date. In addition, engagement may differ in the context of a study compared with the use outside of the study context. However, the consistency between our findings and those of previous research supports the validity of our usability and acceptability findings. Finally, our 2-week follow-up period was relatively short, and user engagement has been shown to decay with time. However, users of quitSTART sustained their use for these 2 weeks, indicating a promising level of initial usability and acceptability during that period.

### Conclusions

Overall, we found that both NCI’s smoking cessation apps (QuitGuide and quitSTART) were usable and appealing among young adults with SMI. However, engagement with quitSTART was high, and ratings of its usability improved with time, indicating that quitSTART may be a more favorable tool than QuitGuide for young adult smokers with SMI. Our findings suggest a possible role for quitSTART during quit attempts in this group; however, clinical support or coaching may be needed to overcome initial usability issues. These findings may assist with the development and adaptation of interventions for young adults with SMI.

## Appendix

Multimedia Appendix 1 Features included in the feature preference task.

Multimedia Appendix 2 Usability tasks.

Multimedia Appendix 3 App feature preferences.

## Abbreviations

CO: carbon monoxide
NCI: National Cancer Institute
ppm: parts per million
SMI: serious mental illness
SUS: System Usability Scale

## References

[1] Smith PH, Mazure CM, McKee SA (2014). Smoking and mental illness in the U.S. population. Tob Control.

[2] Wang TW, Asman K, Gentzke AS, Cullen KA, Holder-Hayes E, Reyes-Guzman C, Jamal A, Neff L, King BA (2018). Tobacco product use among adults - United States, 2017. MMWR Morb Mortal Wkly Rep.

[3] Anthenelli RM, Benowitz NL, West R, Aubin LS, McRae T, Lawrence D, Ascher J, Russ C, Krishen A, Evins AE (2016). Neuropsychiatric safety and efficacy of varenicline, bupropion, and nicotine patch in smokers with and without psychiatric disorders (EAGLES): a double-blind, randomised, placebo-controlled clinical trial. Lancet.

[4] Doll R, Peto R, Boreham J, Sutherland I (2004). Mortality in relation to smoking: 50 years' observations on male British doctors. Br Med J.

[5] Jha P, Ramasundarahettige C, Landsman V, Rostron B, Thun M, Anderson RN, McAfee T, Peto R (2013). 21st-century hazards of smoking and benefits of cessation in the United States. N Engl J Med.

[6] Villanti AC, McKay HS, Abrams DB, Holtgrave DR, Bowie JV (2010). Smoking-cessation interventions for U.S. young adults: a systematic review. Am J Prev Med.

[7] Suls JM, Luger TM, Curry SJ, Mermelstein RJ, Sporer AK, An LC (2012). Efficacy of smoking-cessation interventions for young adults: a meta-analysis. Am J Prev Med.

[8] Brunette MF, Ferron JC, Robinson D, Coletti D, Geiger P, Devitt T, Klodnick V, Gottlieb J, Xie H, Greene MA, Ziedonis D, Drake RE, McHugo GJ (2018). Brief web-based interventions for young adult smokers with severe mental illnesses: a randomized, controlled pilot study. Nicotine Tob Res.

[9] Prochaska JJ, Fromont SC, Ramo DE, Young-Wolff KC, Delucchi K, Brown RA, Hall SM (2015). Gender differences in a randomized controlled trial treating tobacco use among adolescents and young adults with mental health concerns. Nicotine Tob Res.

[10] Brunette MF, Achtyes E, Pratt S, Stilwell K, Opperman M, Guarino S, Kay-Lambkin F (2019). Use of smartphones, computers and social media among people with SMI: opportunity for intervention. Community Ment Health J.

[11] Hoeppner BB, Hoeppner SS, Seaboyer L, Schick MR, Wu GW, Bergman BG, Kelly JF (2016). How smart are smartphone apps for smoking cessation? A content analysis. Nicotine Tob Res.

[12] Gowarty MA, Kung NJ, Maher AE, Longacre MR, Brunette MF (2020). Perceptions of mobile apps for smoking cessation among young people in community mental health care: qualitative study. JMIR Form Res.

[13] Vilardaga R, Casellas-Pujol E, McClernon JF, Garrison KA (2019). Mobile applications for the treatment of tobacco use and dependence. Curr Addict Rep.

[14] Ferron JC, Brunette MF, Geiger P, Marsch LA, Adachi-Mejia AM, Bartels SJ (2017). Mobile phone apps for smoking cessation: quality and usability among smokers with psychosis. JMIR Hum Factors.

[15] Haskins BL, Lesperance D, Gibbons P, Boudreaux ED (2017). A systematic review of smartphone applications for smoking cessation. Transl Behav Med.

[16] Robinson CD, Seaman EL, Grenen E, Montgomery L, Yockey RA, Coa K, Prutzman Y, Augustson E (2020). A content analysis of smartphone apps for adolescent smoking cessation. Transl Behav Med.

[17] Ubhi HK, Michie S, Kotz D, van Schayck OC, Selladurai A, West R (2016). Characterising smoking cessation smartphone applications in terms of behaviour change techniques, engagement and ease-of-use features. Transl Behav Med.

[18] Abroms LC, Westmaas JL, Bontemps-Jones J, Ramani R, Mellerson J (2013). A content analysis of popular smartphone apps for smoking cessation. Am J Prev Med.

[19] Thornton L, Quinn C, Birrell L, Guillaumier A, Shaw B, Forbes E, Deady M, Kay-Lambkin F (2017). Free smoking cessation mobile apps available in Australia: a quality review and content analysis. Aust N Z J Public Health.

[20] Brunette MF, Ferron JC, Devitt T, Geiger P, Martin WM, Pratt S, Santos M, McHugo GJ (2012). Do smoking cessation websites meet the needs of smokers with severe mental illnesses?. Health Educ Res.

[21] Rotondi AJ, Sinkule J, Haas GL, Spring MB, Litschge CM, Newhill CE, Ganguli R, Anderson CM (2007). Designing websites for persons with cognitive deficits: design and usability of a psychoeducational intervention for persons with severe mental illness. Psychol Serv.

[22] Vilardaga R, Rizo J, Ries RK, Kientz JA, Ziedonis DM, Hernandez K, McClernon FJ (2019). Formative, multimethod case studies of learn to quit, an acceptance and commitment therapy smoking cessation app designed for people with serious mental illness. Transl Behav Med.

[23] Andersen M, Perrin A (2017). Tech Adoption Climbs Among Older Adults. Pew Research Center.

[24] Usability.gov: Improving the User Experience.

[25] Bricker JB, Sridharan V, Zhu Y, Mull KE, Heffner JL, Watson NL, McClure JB, Di C (2018). Trajectories of 12-month usage patterns for two smoking cessation websites: exploring how users engage over time. J Med Internet Res.

[26] Strecher VJ, McClure J, Alexander G, Chakraborty B, Nair V, Konkel J, Greene S, Couper M, Carlier C, Wiese C, Little R, Pomerleau C, Pomerleau O (2008). The role of engagement in a tailored web-based smoking cessation program: randomized controlled trial. J Med Internet Res.

[27] Richardson A, Graham AL, Cobb N, Xiao H, Mushro A, Abrams D, Vallone D (2013). Engagement promotes abstinence in a web-based cessation intervention: cohort study. J Med Internet Res.

[28] SRNT Subcommittee on Biochemical Verification (2002). Biochemical verification of tobacco use and cessation. Nicotine Tob Res.

[29] Nielsen J, Landauer TK (1993). A Mathematical Model of the Finding of Usability Problems. CHI '93 Conference on Human Factors in Computing Systems.

[30] Suresh K (2011). An overview of randomization techniques: an unbiased assessment of outcome in clinical research. J Hum Reprod Sci.

[31] van Someren MW, Barnard YF, Sandberg JAC (1994). The Think Aloud Method: A Practical Guide to Modelling Cognitive Processes.

[32] Fiore MC, Jaén CR, Baker TB (2008). Treating Tobacco Use and Dependence: 2008 Update. Clinical Practice Guideline.

[33] Twyman L, Bonevski B, Paul C, Bryant J (2014). Perceived barriers to smoking cessation in selected vulnerable groups: a systematic review of the qualitative and quantitative literature. BMJ Open.

[34] Heatherton TF, Kozlowski LT, Frecker RC, Fagerström KO (1991). The fagerström test for nicotine dependence: a revision of the fagerström tolerance questionnaire. Br J Addict.

[35] Weinberger AH, Reutenauer EL, Allen TM, Termine A, Vessicchio JC, Sacco KA, Easton CJ, McKee SA, George TP (2007). Reliability of the fagerström test for nicotine dependence, Minnesota nicotine withdrawal scale, and Tiffany questionnaire for smoking urges in smokers with and without schizophrenia. Drug Alcohol Depend.

[36] Heffner JL, Vilardaga R, Mercer LD, Kientz JA, Bricker JB (2015). Feature-level analysis of a novel smartphone application for smoking cessation. Am J Drug Alcohol Abuse.

[37] Oliver JA, Hallyburton MB, Pacek LR, Mitchell JT, Vilardaga R, Fuemmeler BF, McClernon FJ (2018). What do smokers want in a smartphone-based cessation application?. Nicotine Tob Res.

[38] Rubin J, Chisnell D (2008). Handbook of Usability Testing: How to Plan, Design, and Conduct Effective Tests.

[39] Brooke J, Thomas B, Jordan PW, McClelland IL, Weerdmeester B (1996). SUS - a quick and dirty usability scale. Usability Evaluation in Industry.

[40] Vilardaga R, Rizo J, Kientz JA, McDonell MG, Ries RK, Sobel K (2016). User experience evaluation of a smoking cessation app in people with serious mental illness. Nicotine Tob Res.

[41] Vilardaga R, Rizo J, Palenski PE, Mannelli P, Oliver JA, Mcclernon FJ (2020). Pilot randomized controlled trial of a novel smoking cessation app designed for individuals with co-occurring tobacco use disorder and serious mental illness. Nicotine Tob Res.

[42] Bangor A, Kortum P, Miller J (2009). Determining what individual SUS scores mean: adding an adjective rating scale. J Usability Stud.

[43] Sauro J (2011). A Practical Guide to the System Usability Scale: Background, Benchmarks & Best Practices.

[44] Lewis JR (2016). Psychometric Evaluation of the Post-Study System Usability Questionnaire: The PSSUQ. Proceedings of the Human Factors and Ergonomics Society Annual Meeting.

[45] Lund A (2001). Measuring usability with the USE questionnaire. Usability Interf.

[46] Fortuna KL, Lohman MC, Gill LE, Bruce ML, Bartels SJ (2017). Adapting a psychosocial intervention for smartphone delivery to middle-aged and older adults with serious mental illness. Am J Geriatr Psychiatry.

[47] Smokefree Initiative.

[48] Braun V, Clarke V (2006). Using thematic analysis in psychology. Qual Res Psychol.

[49] Saldana J (2016). The Coding Manual for Qualitative Researchers.

[50] Gallagher KM, Updegraff JA (2012). Health message framing effects on attitudes, intentions, and behavior: a meta-analytic review. Ann Behav Med.

[51] Toll BA, Rojewski AM, Duncan LR, Latimer-Cheung AE, Fucito LM, Boyer JL, O'Malley SS, Salovey P, Herbst RS (2014). 'Quitting smoking will benefit your health': the evolution of clinician messaging to encourage tobacco cessation. Clin Cancer Res.

[52] Moorman M, van den Putte B (2008). The influence of message framing, intention to quit smoking, and nicotine dependence on the persuasiveness of smoking cessation messages. Addict Behav.

[53] Mays D, Niaura RS, Evans WD, Hammond D, Luta G, Tercyak KP (2015). Cigarette packaging and health warnings: the impact of plain packaging and message framing on young smokers. Tob Control.

[54] Lindson N, Klemperer E, Hong B, Ordóñez-Mena JM, Aveyard P (2019). Smoking reduction interventions for smoking cessation. Cochrane Database Syst Rev.

[55] Bricker JB, Mull KE, Kientz JA, Vilardaga R, Mercer LD, Akioka KJ, Heffner JL (2014). Randomized, controlled pilot trial of a smartphone app for smoking cessation using acceptance and commitment therapy. Drug Alcohol Depend.

[56] Bricker JB, Watson NL, Mull KE, Sullivan BM, Heffner JL (2020). Efficacy of smartphone applications for smoking cessation: a randomized clinical trial. JAMA Intern Med.

[57] Hébert ET, Ra CK, Alexander AC, Helt A, Moisiuc R, Kendzor DE, Vidrine DJ, Funk-Lawler RK, Businelle MS (2020). A mobile just-in-time adaptive intervention for smoking cessation: pilot randomized controlled trial. J Med Internet Res.

[58] Iacoviello BM, Steinerman JR, Klein DB, Silver TL, Berger AG, Luo SX, Schork NJ (2017). Clickotine, a personalized smartphone app for smoking cessation: initial evaluation. JMIR Mhealth Uhealth.

[59] Klein P, Lawn S, Tsourtos G, van Agteren J (2019). Tailoring of a smartphone smoking cessation app (kick.It) for serious mental illness populations: qualitative study. JMIR Hum Factors.

[60] Struik LL, Bottorff JL, Baskerville NB, Oliffe J, Crichton S (2019). Comparison of developers' and end-users' perspectives about smoking cessation support through the crush the crave app. JMIR Mhealth Uhealth.

