# Supplementary material for: Usability and Acceptability of Two Smartphone Apps for Smoking Cessation Among Young Adults With Serious Mental Illness: Mixed Methods Study
Source: JMIR Ment Health. 2021 Jul 7;8(7):e26873. doi: 10.2196/26873 (PMC8295834; doi:10.2196/26873)
Supplement: Multimedia Appendix 1 [file mental_v8i7e26873_app1.docx]

## Multimedia Appendix 1. Features included in the feature preference task

1. Allows you to enter a quit date

2. Allows you to enter a quit plan

3. Allows you to record the number of cigarettes you smoke per day

4. Allows you to record things that make you want to smoke

5. Allows you to record when you have the feeling of wanting a cigarette

6. Allows you to enter a location where you typically smoke, and then sends you an alert when you are near one of those locations

7. Provides information on why quitting is good for you

8. Provides information on how to quit

9. Shows you how many cigarettes you have been smoking

10. Shows you how much money you have saved by not smoking

11. Allows you to share progress on social media (such as Facebook, Twitter, Instagram)

12. Provides a link to other resources for quitting (such as a Quit Smoking Website, or a Quitline)

13. Provides distracting games or other tasks that you can use when you have an urge to smoke

14. Provides reminders about taking quit smoking medication

15. Sends motivational messages throughout the day
