# Supplementary material for: Usability and Acceptability of Two Smartphone Apps for Smoking Cessation Among Young Adults With Serious Mental Illness: Mixed Methods Study
Source: JMIR Ment Health. 2021 Jul 7;8(7):e26873. doi: 10.2196/26873 (PMC8295834; doi:10.2196/26873)
Supplement: Multimedia Appendix 2 [file mental_v8i7e26873_app2.docx]

## Multimedia Appendix 2. Usability Tasks

1. Set a new quit date.

2. Tell the app how many cigarettes per day you are smoking.

3. Tell the app that you’re feeling good right now.

4. Tell the app that you want a cigarette right now.

5. Find information on things you can do to help you quit or reduce your smoking. Please read a little bit of what you find out loud.

6. Tell the app that you smoked a cigarette today.

7. Find where there is information on how much you have been smoking (or how many cigarettes you’ve avoided) since you downloaded the app.

8. Find out how you can connect with other people on social media about quitting or reducing smoking.

9. Find where in the app you can take a photo or upload a photo.
