# Supplementary material for: Usability and Acceptability of Two Smartphone Apps for Smoking Cessation Among Young Adults With Serious Mental Illness: Mixed Methods Study
Source: JMIR Ment Health. 2021 Jul 7;8(7):e26873. doi: 10.2196/26873 (PMC8295834; doi:10.2196/26873)
Supplement: Multimedia Appendix 3 [file mental_v8i7e26873_app3.docx]

## Multimedia Appendix 3. App Feature Preferences

Percent of participants who agree or strongly agree that the listed feature is important to help someone quit or cut back on their smoking.
